# NQR as a target for new antibiotics

**DOI:** 10.3389/fmicb.2025.1690572

**Published:** 2025-11-24

**Authors:** Martín A. González-Montalvo, Jennifer M. Sorescu, Ming Yuan, Joseph DePaolo-Boisvert, Pingdong Liang, Oscar X. Juárez, Karina Tuz

**Affiliations:** 1 Department of Biological Sciences, Illinois Institute of Technology, Chicago, IL, United States; 2 Department of Chemistry, Illinois Institute of Technology, Chicago, IL, United States; 3 Department of Cell and Molecular Biology, Northwestern University, Chicago, IL, United States

**Keywords:** NQR, antimicrobial resistance, respiratory chain, *Vibrio cholerae*, *Pseudomonas aeruginosa*, korormicin, PEG-2S, clofazimine

## Abstract

The rise in antimicrobial resistance has underscored the urgent need for identification of novel targets against antibiotic resistant bacteria, which pose enormous threats to public health. The respiratory enzyme NQR carries essential roles in pathogenic bacteria, producing an ion gradient across the plasma membrane that drives ATP generation by the oxidative phosphorylation system. The vital role of NQR in a multitude of pathogenic microbes for which drug development is a high priority, such as *Vibrio cholerae, Chlamydia trachomatis*, and *Pseudomonas aeruginosa*, makes it an ideal drug target meriting investigation, especially since this enzyme is absent in human cells. A diverse array of NQR inhibitors have previously been identified, ranging from the ubiquinone analogs korormicin, HQNO, and aurachin D-42, which occupy one of two ubiquinone binding sites, to monovalent and divalent cations such as Ag^+^ and Zn^2+^ that react with SH groups. To overcome cytotoxicity associated with many established NQR inhibitors, drug development efforts have produced synthetic analogs of korormicin that exhibit minimal toxicity. To address the urgent need for alternative treatments, our group has explored the repurposing of FDA-approved drugs with established safety profiles as NQR inhibitors. Our recent work revealed that clofazimine, and FDA-approved orphan drug, is as a potent NQR inhibitor with strong antivirulence properties. This review highlights the role and significance of NQR and its inhibitors, with an emphasis on the potential development of antibiotics to target this respiratory enzyme.

## 1 Introduction

### 1.1 Antibiotic resistance

The world is currently facing a costly, silent and dangerous pandemic: antimicrobial resistance (AMR). The lack of proper antibiotic stewardship in both clinical and agricultural settings has contributed to the widespread emergence, dissemination, and persistence of multidrug-resistant (MDR) organisms (Endale et al., 2023; Manyi-Loh et al., 2018; Nardulli et al., 2022; Van Boeckel et al., 2015; Williams-Nguyen et al., 2016; Xi et al., 2009; Zhang et al., 2009). This threat has grown into a global crisis and in 2019 alone, antibiotic-resistant infections were estimated to have caused around 5 million deaths (Murray et al., 2022). By 2050, it is projected that more than 10 million deaths will occur yearly (Naghavi et al., 2024). Moreover, in the next 25 years, these infections will likely produce 200 million deaths, including direct and AMR associated deaths (Naghavi et al., 2024). Despite this escalating threat, antibiotic development remains focused on a limited number of bacterial targets: DNA replication, protein synthesis, cell wall synthesis, and membrane integrity (Belete, 2019; Brown and Wright, 2016; Coates et al., 2011; World Health Organization, 2024a). Unfortunately, the pipeline for new antibiotics and therapeutic options remains limited, as pathogens show an alarming ability to quickly develop resistance to antimicrobials (Coates et al., 2011; World Health Organization, 2024c). Given this critical situation, identifying novel approaches and targets to combat MDR infections is urgently needed.

In the past decade, the bacterial respiratory metabolism has emerged as a promising area for drug development (Bald and Koul, 2010; Gaupp et al., 2010; González-Montalvo et al., 2024; Grauel et al., 2021; Heikal et al., 2014; Hu et al., 2024; Lencina et al., 2018; Liang et al., 2020, 2018; Mogi et al., 2009; Raba et al., 2018; Radloff et al., 2021; Schurig-Briccio et al., 2020; Van Alst et al., 2022). Respiratory chains play critical roles in pathogen physiology, supporting the entire metabolic network and transport activities throughout the plasma membrane, which are crucial to maintaining homeostatic processes and growth (Kaila and Wikström, 2021). Due to their fundamental roles in pathogens, respiratory chain enzymes are highly attractive pharmacologic targets. This was first demonstrated in *Mycobacterium tuberculosis* with the introduction of bedaquiline, a drug that targets the terminal step of oxidative phosphorylation, inhibiting the F_1_–F_0_ ATP synthase (Cox and Laessig, 2014). Bedaquiline was rapidly approved for the treatment of MDR tuberculosis as part of a combination therapy, despite its potential for serious side effects, highlighting the urgent need for alternative treatments in patients who suffer from MDR infections and often face poor prognoses (Cox and Laessig, 2014; Field, 2015; U.S. Food and Drug Administration, 2013). Recently, other enzymes in the oxidative phosphorylation pathway, particularly components of the electron transport chain, have become the focus of intense research. Special attention has been given to bacterial respiratory enzymes that differ significantly from their mammalian counterparts (Balemans et al., 2012; Cook et al., 2014; Grauel et al., 2021; Hards and Cook, 2018; Lencina et al., 2018). Among the most studied targets are respiratory NADH dehydrogenases, whose inhibition or deletion impairs growth and/or virulence in several pathogens, including *Staphylococcus aureus* (Schurig-Briccio et al., 2020), *Chlamydia trachomatis* (Liang et al., 2018; Stephens et al., 1998)*, Pseudomonas aeruginosa* (Hreha et al., 2021), and *Vibrio cholerae* (Agarwal et al., 2020; Minato et al., 2014).

This review focuses on the NQR respiratory complex, a H^+^/ Na^+^-translocating NADH:quinone oxidoreductase, which is widely distributed across the bacterial domain (Juarez and Barquera, 2012; Reyes-Prieto et al., 2014). NQR has recently attracted interest both as an antibiotic and as an antivirulence target for the treatment of MDR infections. NQR is essential to bacterial physiology, supporting energy generation, maintaining membrane potential, and driving transport systems critical for growth and survival. Due to its central role, NQR represents a highly attractive pharmacological target. In *V. cholerae*, NQR has been shown to be essential for the expression and production of virulence factors (Häse and Mekalanos, 1999; Minato et al., 2014; Toulouse et al., 2018). Furthermore, it has been reported that mutants lacking NQR are completely avirulent (Minato et al., 2014). Because of its fundamental importance in pathogenic bacteria and its absence in mammalian cells, this complex is a promising target for drug design (Dibrov et al., 2017). This manuscript showcases recent advances in developing antibiotics and antivirulence strategies that target NQR, aiming to expand our arsenal against multidrug-resistant pathogens.

### 1.2 NQR structure and composition

**FIGURE 1:**
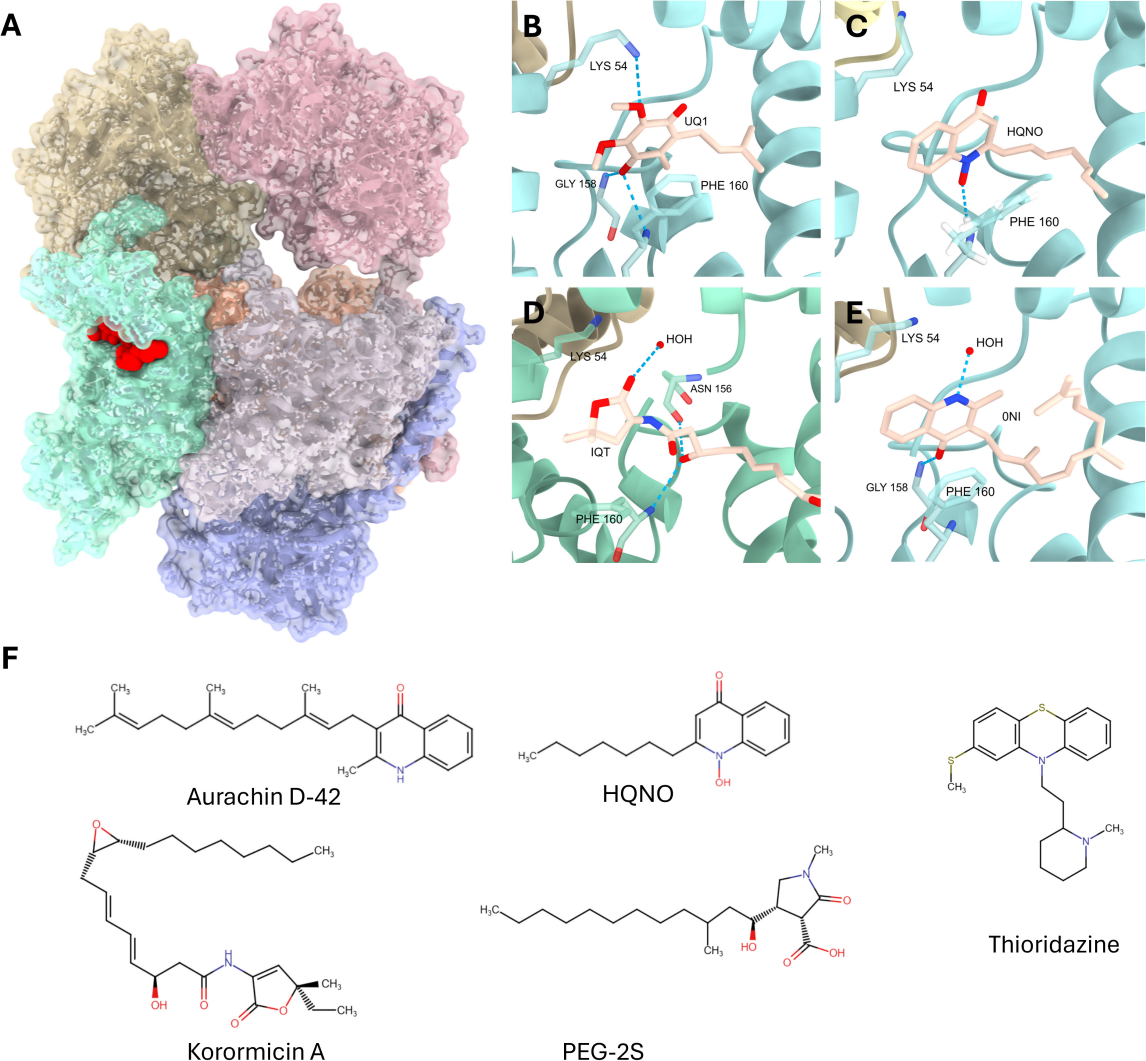
**(A)** Tri-dimensional structure of *V. cholerae* NQR in complex with korormicin (PDBID: 7XK7) (Kishikawa et al., 2022). Protein—Beige: NqrA, Teal: NqrB, Blue: NqrC, Gray: NqrD, Orange: NqrE, Pink: NqrF. Spheres—Red: Korormicin. **(B–E)** Close-up views of the ubiquinone binding pocket with ubiquinone-1 (UQ1), HQNO, korormicin (IQT), and aurachin D-42 (0NI) bound. Structures were obtained from PDB-IDS 8EVU (Juarez and Fuller, 2022), 8A1Y (Hau et al., 2023), 7XK7 (Kishikawa et al., 2022), and 7XK6 (Kishikawa et al., 2022), respectively. **(F)** NQR inhibitors described in this review: aurachin D-42, korormicin A, 2-n-heptyl-4-hydroxyquinoline-N-oxide (HQNO), PEG-2S, clofazimine, and thioridazine.

NQR is a membrane-bound enzyme complex found in many bacteria (Juarez and Barquera, 2012; Reyes-Prieto et al., 2014), particularly marine and pathogenic species such as *V. cholerae* (Häse and Mekalanos, 1998; Lin et al., 2007; Toulouse et al., 2018; Yuan et al., 2025), *C. trachomatis* (Liang et al., 2018; Stephens et al., 1998), *P. aeruginosa* (Liang et al., 2020; Raba et al., 2018), *Klebsiella* spp. (Fadeeva et al., 2007; González-Montalvo et al., 2024), and many others (Reyes-Prieto et al., 2014). NQR plays a critical role during infection and multiplication by transferring electrons from NADH to the quinone pool. During this process, NQR transports sodium ions (Dimroth, 1997; Juárez et al., 2009) or protons (Raba et al., 2018) from the cytosol to the periplasmic space, generating a sodium or proton motive force. This gradient is vital for various cellular functions, including ATP synthesis, nutrient uptake, flagellar rotation, and ion homeostasis (Dashper et al., 2001; Häse and Barquera, 2001; Huda et al., 2001; Reyes-Prieto et al., 2014; von Ballmoos et al., 2002). By contributing to energy metabolism, ion homeostasis and environmental adaptation, NQR supports pathogen survival, colonization, and virulence. NQR is a respiratory complex composed of six subunits, NqrA-F (Figure 1A). The complex contains several cofactors that facilitate the transfer of electrons through the enzyme, including an FAD and a 2Fe-2S cluster center located in NqrF (Barquera et al., 2004); one 2Fe-2S cluster located between subunits NqrD and NqrE (Kishikawa et al., 2022); two FMNs attached through covalent bonds to subunits NqrC and NqrB (Barquera et al., 2006); and a riboflavin molecule non-covalently bound to NqrB (Barquera et al., 2002; Tuz et al., 2022). Interestingly, NQR and its close relative RNF are the only reported enzymes that utilize riboflavin as a redox cofactor (Juárez et al., 2008; Vitt et al., 2022). The first tri-dimensional structures of *V. cholerae* NQR were deposited to the Protein Data Bank in 2014 (Berman, 2000; Steuber et al., 2014). These included sub-2Å resolution structures of the water-soluble subunits (NqrA, NqrC, NqrF) and a 3.5 Å structure of the complete complex. Since then, 21 structures of *V. cholerae* NQR have been deposited in complex with the native substrates and inhibitory compounds including HQNO, aurachin-D, and korormicin (Hau et al., 2023; Kishikawa et al., 2022). Our group utilized an *in silico* approach to analyze the binding of these compounds to *V. cholerae* NQR by Molecular Dynamics Simulation. These simulations demonstrate residue-specific interactions between NqrB and inhibitors, actionable in the formation of structure-activity-relationships for the development of novel NQR inhibitors (DePaolo-Boisvert et al., 2025). Our molecular docking data is consistent with available structural data and reveals other transient interactions that could be exploited pharmacologically, highlighting the importance of this *in silico* tool in drug discovery, especially for NQR.

## 2 Target validation in pathogenic bacteria

### 2.1 Vibrio cholerae

*V. cholerae* is a Gram-negative intestinal pathogen causing cholera, a diarrheal disease that is life-threating in absence of prompt therapy (Montero et al., 2023; Ojeda Rodriguez et al., 2025). It is estimated that *V. cholerae* causes up to 4 million cases per year and 20,000–140,000 deaths worldwide (Ali et al., 2015; World Health Organization, 2020). *V. cholerae* remains a significant threat due to its capacity to produce pandemics, as well as the emergence and spread of multi-drug resistant strains (Rijal et al., 2019; Smith et al., 2015; Thapa Shrestha et al., 2015). The *V. cholerae* respiratory chain is relatively simple compared to other bacteria (Bueno et al., 2020; Steuber et al., 2015), with NQR playing a major role in sodium homeostasis (Häse and Barquera, 2001; Häse and Mekalanos, 1999; Lin et al., 2007). NQR is the main entry point of electrons into the respiratory chain (Agarwal et al., 2020; Häse and Barquera, 2001; Lin et al., 2007; Steuber and Fritz, 2024). Several reports show that NQR is critical for *V. cholerae* virulence, particularly for colonization and stomach acid tolerance (Merrell et al., 2002; Minato et al., 2014). NQR inactivation or inhibition impairs the expression of virulence factors, specifically the toxin-coregulated pilus (TCP) and the cholera toxin (CT) (Merrell et al., 2002; Minato et al., 2014). Remarkably, NQR activity regulates the expression of the ToxT regulator, which controls the transcription of CT and TCP (Häse and Mekalanos, 1999). It has been reported that Δ*nqr* mutants secrete significantly less CT in culture (Häse and Mekalanos, 1999; Minato et al., 2014). In a similar manner, the autoagglutinating phenotype of *V. cholerae*, which is dependent on TCP expression, is reduced in the NQR mutant (Minato et al., 2014). The regulatory mechanism of toxicity has been linked specifically to NQR and its role in the respiratory chain, since deletion of other sodium pumps has no effect on virulence (Minato et al., 2014). This evidence supports the role of NQR as a vital enzyme for *V. cholerae* during infection, making this respiratory complex an attractive target for drug development. Inhibiting this respiratory enzyme may not only promote clearing of the bacteria but may also reduce its virulence, attenuating infection severity.

### 2.2 Chlamydia trachomatis

*C. trachomatis* is a Gram-negative, obligate intracellular pathogen responsible for trachoma (eye infection) and chlamydia (genital infection) (Burton and Mabey, 2009; Elwell et al., 2016; Huai et al., 2020). The developmental cycle of *C. trachomatis* is composed of two forms, the infectious and metabolically inactive elementary body, and the non-infectious but metabolically active reticulate body (Bayramova et al., 2018; Elwell et al., 2016). Genomic analyses indicate that despite its reduced genome, *C. trachomatis* possesses a simplified, but functional respiratory chain composed of NQR, succinate dehydrogenase, cytochrome *bd* oxidase, and a sodium-dependent A_1_-A_0_ type ATP synthase (Dibrov et al., 2004; Liang et al., 2018; Stephens et al., 1998). Among these enzymes, NQR plays a central role by coupling NADH oxidation to menaquinone reduction while simultaneously pumping sodium across the bacterial inner membrane (Barta et al., 2014; Juárez et al., 2010), which generates a gradient that is utilized to drive ATP synthesis, providing *C. trachomatis* with a host-independent means of energy production. Our group reported that *C. trachomatis* has a high respiratory activity resistant to mitochondrial inhibitors but sensitive to NQR inhibitors (Liang et al., 2018). Functional assays further revealed that inhibiting NQR or collapsing the sodium gradient with ionophores severely impairs chlamydial inclusion development and reduces bacterial protein expression without significantly disrupting host mitochondrial function (Liang et al., 2018). These effects were most pronounced in reticulate body-infected cells, in which the pathogen is rapidly multiplying. Additionally, *in vitro* studies with permeabilized cells showed that chlamydial respiration is stimulated by α-ketoglutarate and ADP, resembling classical state three mitochondrial respiration (Liang et al., 2018; Meister, 2009). Altogether, these findings establish that NQR is a critical component of a sodium-driven respiratory chain in *C. trachomatis*, supporting ATP production and intracellular replication. This sodium-based oxidative phosphorylation pathway challenges the long-standing view of *C. trachomatis* as an obligate energy parasite (Dibrov et al., 2004; Häse et al., 2001). Moreover, this system represents a unique metabolic adaptation of *Chlamydia* and a potential therapeutic target for disrupting chlamydial energy homeostasis.

### 2.3 Pseudomonas aeruginosa

*P. aeruginosa* is a Gram-negative bacterium that is a leading cause of mortality in immunocompromised patients and those suffering from chronic lung diseases (Bhagirath et al., 2016; Centers for Disease Control and Prevention, 2022). The World Health Organization classified carbapenem-resistant *P. aeruginosa* as a high priority pathogen, highlighting the pressing need for novel antibiotic development against this bacterium (World Health Organization, 2024b). In *P. aeruginosa*, the NQR complex is distinct among other NQR homologs, as it is a proton pump (Raba et al., 2018) fostering an energy-generating gradient across the cell membrane (Raba et al., 2018; Juarez and Barquera, 2012; Reyes-Prieto et al., 2014). NQR is the primary NADH dehydrogenase employed by *P. aeruginosa* in physiologically relevant modified artificial urinary media and LB broth, responsible for more than 75% of electron transfer in both stationary and logarithmic growth phases (Liang et al., 2020; Hreha et al., 2021; Hu et al., 2024). Mutant analysis confirmed the importance of NQR, as approximately half of *P. aeruginosa* NADH dehydrogenase activity was halted in both growth phases in the mutant of this enzyme (Hreha et al., 2021). Despite its critical role, deletion of the enzyme was not shown to impact growth *in vitro* (Torres et al., 2019), indicating that other dehydrogenases can be used by this bacterium to compensate. Further evaluation of NQR inhibition on *P. aeruginosa* virulence is necessary, as its role in the electron transport chain in media simulating physiological and infection site conditions proved significant (Hu et al., 2024). Therefore, NQR seems to be arising as a promising target to treat MDR infections caused by *P. aeruginosa*, providing a much necessary alternative to current therapeutic options.

## 3 Reported NQR inhibitors

### 3.1 Ubiquinone analogs

Several compounds have been identified as NQR inhibitors, with different potencies and action mechanisms. For example, korormicin (Figure 1F), an antibiotic produced by *Pseudoalteromonas* sp. F-420, has inhibition against *V. cholerae* NQR, blocking its activity with extremely high potency (IC_50_ of 5 nM) while simultaneously hindering the ability of NQR to pump sodium (Ito et al., 2017; Yoshikawa et al., 1999; Table 1). Enzymatic analysis shows that it has mixed inhibition (originally described as non-competitive) in relation to ubiquinone (Hayashi et al., 2002), indicating that it binds to the ubiquinone site but that it might have another inhibition site (Figures 1B–D). Additionally, bacterial secondary metabolites, including 2-n-heptyl-4-hydroxyquinoline-*N*-oxide (HQNO) (Figure 1F) from *P. aeruginosa* and aurachin D-42 (Figure 1F) derived from the myxobacteria *Stigmatella aurantiaca*, are compounds that mimic ubiquinone and have also demonstrated efficacy in inhibiting NQR activity (Ito et al., 2017; Kunze et al., 1987; Tokuda and Unemoto, 1984) by binding to the ubiquinone site as well (Figures 1B,C,E and Table 1). HQNO was considerably less potent than aurachin D-42, requiring concentrations exceeding 100 nM to achieve 50% inhibition of *V. alginolyticus* NQR and 2.1 μM for *V. cholerae* NQR, whereas the highly potent aurachin D-42 has an IC_50_ of 2 nM against *V. cholerae* NQR (Ito et al., 2017; Yoshikawa et al., 1999; Table 1). Interestingly, *P. aeruginosa* is resistant to HQNO due to variations in amino acids at the ubiquinone-binding site of subunit D (Raba et al., 2018), suggesting that structural differences in Pa-NQR provide an advantage against similar inhibitor molecules.

**TABLE 1:** Reported NQR inhibitors.

Inhibitor	IC_50_	Microorganism	Inhibition mechanism
HQNO	2.1 μM (Masuya et al., 2020)	*V. cholerae*	Binds to ubiquinone site(s) (Tuz et al., 2015)
Korormicin	5 nM (Ito et al., 2017)	*V. cholerae*	Noncompetitive inhibitor at ubiquinone site (Masuya et al., 2020)
PEG-2S	1.8 nM (Dibrov et al., 2017)	*V. cholerae, C. trachomatis*	Likely similar to korormicin
Aurachin D-42	2 nM (Ito et al., 2017)	*V. cholerae*	Binds to ubiquinone site (Masuya et al., 2020)
Ag^+^	20 nM (Hayashi et al., 1992)	*V. alginolyticus*	Binds cysteine residues in NqrF; disrupts FAD/NADH sites and quinone binding pocket (Fadeeva et al., 2011, 2008)
Zn^2+^	1 μM (Hayashi et al., 1992)	*V. alginolyticus*	Reacts with thiol groups in NqrF (Hayashi et al., 1992)
Clofazimine	3 μM (Yuan et al., 2025)	*V. cholerae*	Non-competitive vs. ubiquinone. Likely binds to the catalytic site in subunits B and D.
Thioridazine	22 μM (Yuan et al., 2025)	*V. cholerae*	Non-competitive vs. ubiquinone

Recently, NQR structures have been solved by cryo-electron microscopy with the substrates ubiquinone-1 and ubiquinone-2, as well as the inhibitors korormicin (Kishikawa et al., 2022), HQNO (Hau et al., 2023), and aurachin D-42 (Kishikawa et al., 2022). Ubiquinone and the inhibitors are bound in a membrane-embedded pocket, principally comprised by NqrB with some contribution by NqrA (Figure 1). This same pocket has been identified in structures 8EVU, 8A1U, 8A1V, and 8A1W (Hau et al., 2023) to carry a tightly-bound ubiquinone (Juarez et al., 2012; Tuz et al., 2022). Although NQR bears a second ubiquinone binding site at the cleft of subunits NqrB and NqrD (Juarez et al., 2012), the exact function and relationship between these two sites is not yet well understood. In an *in silico* study (Dibrov et al., 2022), the catalytic ubiquinone site was modeled in the membrane, however this model is limited since it only examined the NqrB-NqrD subunits, lacking major parts of the context. Regarding the site that has been solved, the residue triad NqrB-G158, F159, and F160 comprise a helix turn responsible for multiple non-covalent interactions between NQR and these ligands. Ubiquinone, korormicin, HQNO, and aurachin D-42 can each be observed accepting one or more hydrogen bonds donated by the backbone nitrogen of this triad. Additionally, NqrB-F160 forms π-stacking interactions (8A1Y, HQNO) and cation-π interactions (7XK6, aurachin D-42) with ligands. Other residues can also be observed establishing less frequent interactions with these ligands. NqrB-K54 and NqrB-N156 are each observed donating a hydrogen bond to acceptors on ubiquinone (8EVU) and korormicin (7XK7).

### 3.2 Monovalent and divalent cations

Inhibitors of NQR that bear no structural resemblance to the ubiquinone substrate, such as metal ions, have also been identified. Ag^+^ and Zn^2+^ ions inhibit the NADH dehydrogenase activity of NQR in some organisms, ultimately disrupting electron transfer activity and ion translocation, hindering ATP generation (Engelking, 2015; Hayashi et al., 1992). In *Vibrio harveyi*, Ag^+^ inhibition of NQR is linked to interactions of these ions with cysteine residues, particularly reactive thiol groups in the NqrF subunit, within the regions responsible for NADH and FAD binding (Fadeeva et al., 2011, 2008). Additionally, NqrF-C377 was identified as relevant for the effects of Ag^+^, Zn^2+^, and other metal ions on NQR activity, while in the oral bacteria *Porphyromonas gingivalis*, the residue NqrF-C383 was considered essential for inhibition (Fadeeva et al., 2011). Ag^+^ exhibits significant potency against *V. alginolyticus* NQR with an IC_50_ of 20 nM (Hayashi et al., 1992; Table 1). Further analysis of Ag^+^ efficacy on *V. alginolyticus* NQR showed that it interacts with the enzyme in the quinone binding site situated in the B subunit, disrupting the enzymatic structure and causing the release of FAD (Nakayama et al., 1999; Unemoto et al., 1993). Zn^2+^ is less potent than Ag^+^, with complete inhibition of *V. alginolyticus* NQR at 3 μM and an IC_50_ of 1 μM (Hayashi et al., 1992; Table 1). Studying these metal ions as inhibitors offers important insights for the development of antimicrobial agents aimed at targeting this critical enzyme.

### 3.3 Furanones

Although several NQR inhibitors have been described, these molecules have significant toxicity to mammalian cells (Dejon and Speicher, 2013; Dibrov et al., 2017; Mo et al., 2023). Recently, a less toxic korormicin-like furanone compound, PEG-2S (Figure 1F), was developed and produced as a pure stereoisomer (Dibrov et al., 2017). In this compound, the epoxy group found in the hydrophobic tail of the korormicin molecule was removed, which was believed to be the source of the toxicity. Moreover, the aliphatic chain was shortened in an attempt to reduce hydrophobicity and increase its potency (Dibrov et al., 2017). The data show that PEG-2S inhibits NADH oxidase activity in isolated *V. cholerae* membranes with an IC_50_ of 1.8 nM (Dibrov et al., 2017; Table 1). PEG-2S was also able to interfere with *C. trachomatis* metabolism in infected HEK293 cells (Dibrov et al., 2017). This pathogen acidifies the cytoplasm of host cells with a subsequent increase in intracellular Na^+^, which is subverted by addition of PEG-2S (Dibrov et al., 2017). This compound was also able to reduce *C. trachomatis* infection of HeLa cells with 26-fold greater potency than korormicin (Dibrov et al., 2017). A one-time treatment of infected HeLa cells with 700 nM PEG-2S reduced the amount of intracellular inclusions by half, while a two-dose treatment with 1 μM reduced inclusions by 90% (Dibrov et al., 2017). Importantly, at a 20 μM concentration, PEG-2S is non-cytotoxic to primary cell cultures and has no effect on other intestinal bacteria (Dibrov et al., 2017). Unfortunately, direct tests of PEG-2S on *C. trachomatis* NQR have not been performed and it is unclear if NQR is the only or actual target in the cell.

### 3.4 Drug repurposing: clofazimine

Traditional antibiotics are becoming increasingly ineffective, and the high cost and long timelines associated with developing new drugs have left the pipeline for novel antibiotics dangerously sparse (Salam et al., 2023). In response to the growing threat posed by multidrug-resistant bacteria to global health, recent research has focused on the repurposing of FDA-approved drugs with established safety profiles (Aggarwal et al., 2024; Ashburn and Thor, 2004; Liu et al., 2021; Nossier et al., 2025; Peyclit et al., 2019; Tovar-Nieto et al., 2024; Urbina et al., 2021). This strategy could be particularly important for *V. cholerae* infections in developing regions where outbreaks can rapidly overwhelm healthcare systems (Marin et al., 2013). That is the case of phenothiazines and phenazines, which numerous studies have shown that in addition to their antipsychotic impact, can be used as antibiotics (Dastidar et al., 2005; Mazumder et al., 2001; Molnár et al., 1976; Thanacoody, 2007). Our group screened a panel of phenothiazines and phenazines and identified the FDA-approved drug clofazimine (Yuan et al., 2025) (Figure 1F), an orphan drug originally used to treat leprosy and tuberculosis (Arbiser and Moschella, 1995; Gopal et al., 2013), as a potent antibiotic against *V. cholerae*, with strong antivirulence properties (Table 1). We also found that thioridazine (Figure 1F) has potent inhibitory effects against *V. cholerae* growth. Clofazimine showed strong antibiotic activity against the pathogen, with a MIC_50_ of 3.5 μM and an IC_50_ of 3 μM against NQR, meanwhile thioridazine had an MIC_50_ of 27 μM and an IC_50_ of 22 μM (Table 1; Yuan et al., 2025). These compounds were tested on two clinical strains of *V. cholerae*: 2010EL-1786 and 2012EL-2176, which are especially important because they were major contributors to the 2010 Haiti humanitarian crisis (Folster et al., 2014) and have multidrug resistance profiles. These two strains have similar MDR profiles, with 2012EL-2176 showing additional resistance to beta-lactam antibiotics (Folster et al., 2014). Despite their multidrug resistance, our data show that clofazimine has a MIC against *V. cholerae* 2010EL-1786 and 2012EL-2176 in the same range as the lab strain O395 (Yuan et al., 2025), indicating that these strains have not evolved resistance mechanisms for phenazines and that clofazimine could be used to treat pandemic strains. Clofazimine treatment also significantly improved survival in an animal model, with an efficacy comparable to ampicillin, while reducing bacterial colonization and production of the cholera toxin *in vitro* (Yuan et al., 2025).

**FIGURE 2:**
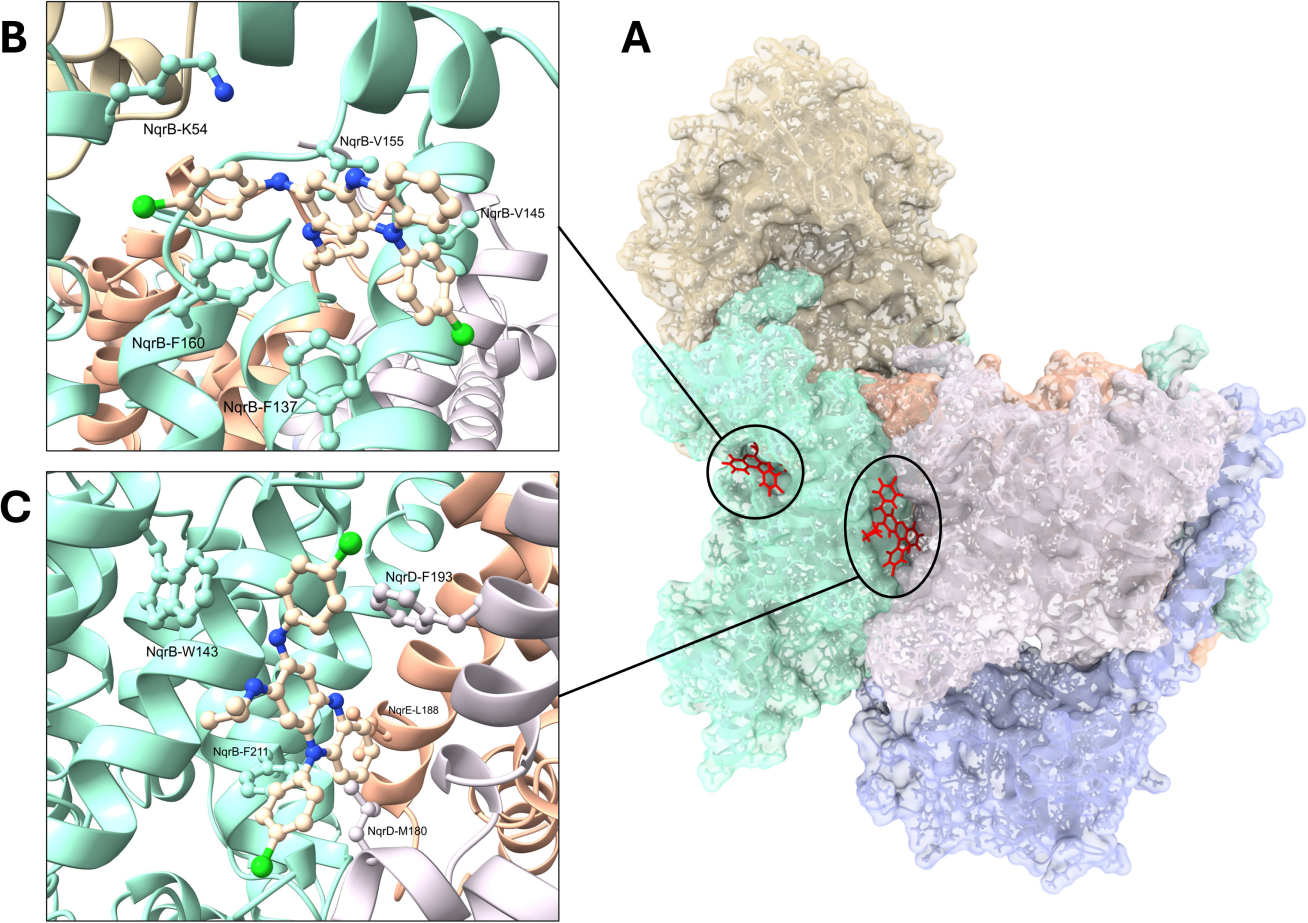
Docked poses of clofazimine to Cryo-EM structure 8EVU. **(A)** Tri-dimensional structure of NQR with clofazimine poses. Protein—Beige: NqrA, Teal: NqrB, Blue: NqrC, Gray: NqrD, Orange: NqrE, Pink: NqrF. Sticks—Red: Clofazimine. **(B,C)** Close-up view of the two docked poses, located, respectively, at the ubiquinone binding site and the NqrB-NqrD cleft.

Our work shows that the main target of clofazimine in *V. cholerae* is the NQR complex (Yuan et al., 2025), demonstrating that NQR is an essential enzyme in the physiology of this bacterium, involved in the generation of the sodium gradient, toxin secretion, motility, and antibiotic resistance (Juarez and Barquera, 2012). Biochemical experiments demonstrated that both clofazimine and thioridazine specifically inhibit the ubiquinone reductase activity of the NQR complex without affecting NADH oxidation, consistent with their mixed inhibition mechanism (Table 1; Yuan et al., 2025). Furthermore, targeted mutations in key residues, such as F211A, decreased drug sensitivity, supporting a direct interaction with the catalytic site of the complex. Molecular docking studies revealed that clofazimine is positioned in the same binding site as natural inhibitors such as HQNO and korormicin (Figure 2). Our work emphasizes the potential of repurposing FDA-approved drugs, such as clofazimine, to treat MDR infections. Clofazimine is a safe, well-characterized drug that can combat cholera and potentially other bacterial infections, especially those involving multidrug-resistant pathogens where NQR is prevalent and functionally indispensable.

## 4 Conclusion

NQR is a unique bacterial respiratory enzyme that is essential for energy metabolism, ion homeostasis, and virulence in a wide range of pathogenic species, including *V. cholerae, C. trachomatis, and P. aeruginosa*. As a respiratory complex absent in mammalian cells, but critical for bacterial survival and pathogenesis, NQR represents a highly attractive target for antimicrobial therapy. Across pathogens, NQR supports intracellular replication, virulence gene expression, and adaptation to host environments through sodium or proton motive force generation. The validation of NQR as essential in various pathogenic contexts confirms its functional importance and potential for broad-spectrum targeting. A variety of inhibitors, including natural products like korormicin and aurachin D-42, synthetic compounds, and divalent cations, demonstrate the vulnerability of NQR to pharmacological disruption. However, early inhibitors have often been limited by toxicity or lack of selectivity. Recent advances, such as the development of PEG-2S, a non-toxic furanone derivative, mark a significant leap forward in the therapeutic exploitation of this enzyme. The data presented here emphasize the centrality of NQR in pathogen physiology and pathogenesis and provide a compelling rationale for the continued development of selective NQR inhibitors as broad-spectrum, host-safe antimicrobials.

Recently, a non-conventional therapeutic option has begun to gain interest: antivirulence therapy. This strategy focuses on developing drugs that reduce the pathogens’ ability to produce virulence determinants instead of killing it directly. Drug development is now expanding beyond essential bacterial processes to include molecular targets that regulate virulence. By disarming rather than eliminating pathogens, antivirulence compounds may offer effective treatment options while reducing selective pressure for resistance, broadening the therapeutic landscape and improving patient outcomes. Clofazimine is an agent that has these characteristics and can be used to reduce the mortality and morbidity in patients with MDR infections, particularly those suffering from MDR *V. cholerae*.

## References

[B1] Agarwal S. Bernt M. Toulouse C. Kurz H. Pfannstiel J. D’Alvise P. (2020). Impact of Na ^+^ -translocating NADH:quinone oxidoreductase on iron uptake and nqrm expression in *Vibrio cholerae*. *J. Bacteriol.* 202:e00681-19. 31712283 10.1128/JB.00681-19 PMC6964743

[B2] Aggarwal M. Patra A. Awasthi I. George A. Gagneja S. Gupta V. (2024). Drug repurposing against antibiotic resistant bacterial pathogens. *Eur. J. Med. Chem.* 279:116833. 10.1016/j.ejmech.2024.116833 39243454

[B3] Ali M. Nelson A. R. Lopez A. L. Sack D. A. (2015). Updated global burden of cholera in endemic countries. *PLoS Negl. Trop. Dis.* 9:e0003832. 10.1371/journal.pntd.0003832 26043000 PMC4455997

[B4] Arbiser J. L. Moschella S. L. (1995). Clofazimine: A review of its medical uses and mechanisms of action. *J. Am. Acad. Dermatol.* 32 241–247. 10.1016/0190-9622(95)90134-5 7829710

[B5] Ashburn T. T. Thor K. B. (2004). Drug repositioning: Identifying and developing new uses for existing drugs. *Nat. Rev. Drug Discov.* 3 673–683. 10.1038/nrd1468 15286734

[B6] Bald D. Koul A. (2010). Respiratory ATP synthesis: The new generation of mycobacterial drug targets?: Respiratory ATP synthesis in mycobacteria. *FEMS Microbiol. Lett.* 308 1–7. 10.1111/j.1574-6968.2010.01959.x 20402785

[B7] Balemans W. Vranckx L. Lounis N. Pop O. Guillemont J. Vergauwen K. (2012). Novel antibiotics targeting respiratory ATP synthesis in gram-positive pathogenic bacteria. *Antimicrob. Agents Chemother.* 56 4131–4139. 10.1128/AAC.00273-12 22615276 PMC3421580

[B8] Barquera B. Nilges M. J. Morgan J. E. Ramirez-Silva L. Zhou W. Gennis R. B. (2004). Mutagenesis study of the 2Fe-2S center and the FAD binding site of the Na(+)-translocating NADH:ubiquinone oxidoreductase from *Vibrio cholerae*. *Biochemistry* 43 12322–12330. 10.1021/bi048689y 15379571

[B9] Barquera B. Ramirez-Silva L. Morgan J. E. Nilges M. J. (2006). A new flavin radical signal in the Na(+)-pumping NADH:quinone oxidoreductase from *Vibrio cholerae*. An EPR/electron nuclear double resonance investigation of the role of the covalently bound flavins in subunits B and C. *J. Biol. Chem.* 281 36482–36491. 10.1074/jbc.M605765200 16973619

[B10] Barquera B. Zhou W. Morgan J. E. Gennis R. B. (2002). Riboflavin is a component of the Na+-pumping NADH-quinone oxidoreductase from *Vibrio cholerae*. *Proc. Natl. Acad. Sci. U. S. A.* 99 10322–10324. 10.1073/pnas.162361299 12122213 PMC124912

[B11] Barta M. L. Thomas K. Yuan H. Lovell S. Battaile K. P. Schramm V. L. (2014). Structural and biochemical characterization of *Chlamydia trachomatis* hypothetical protein CT263 supports that menaquinone synthesis occurs through the futalosine pathway. *J. Biol. Chem.* 289 32214–32229. 10.1074/jbc.M114.594325 25253688 PMC4231696

[B12] Bayramova F. Jacquier N. Greub G. (2018). Insight in the biology of chlamydia-related bacteria. *Microbes Infect.* 20 432–440. 10.1016/j.micinf.2017.11.008 29269129

[B13] Belete T. M. (2019). Novel targets to develop new antibacterial agents and novel alternatives to antibacterial agents. *Hum. Microb. J.* 11:100052. 10.1016/j.humic.2019.01.001

[B14] Berman H. M. (2000). The protein data bank. *Nucleic Acids Res.* 28 235–242. 10.1093/nar/28.1.235 10592235 PMC102472

[B15] Bhagirath A. Y. Li Y. Somayajula D. Dadashi M. Badr S. Duan K. (2016). Cystic fibrosis lung environment and *Pseudomonas aeruginosa* infection. *BMC Pulm Med.* 16:174. 10.1186/s12890-016-0339-5 27919253 PMC5139081

[B16] Brown E. D. Wright G. D. (2016). Antibacterial drug discovery in the resistance era. *Nature* 529 336–343. 10.1038/nature17042 26791724

[B17] Bueno E. Pinedo V. Cava F. (2020). Adaptation of *Vibrio cholerae* to hypoxic environments. *Front. Microbiol.* 11:739. 10.3389/fmicb.2020.00739 32425907 PMC7212424

[B18] Burton M. J. Mabey D. C. W. (2009). The global burden of trachoma: A review. *PLoS Negl. Trop. Dis.* 3:e460. 10.1371/journal.pntd.0000460 19859534 PMC2761540

[B19] Centers for Disease Control and Prevention (2022). *COVID-19: U.S. impact on antimicrobial resistance, special report 2022.* Atlanta, GA: Centers for Disease Control and Prevention, 10.15620/cdc:117915

[B20] Coates A. R. Halls G. Hu Y. (2011). Novel classes of antibiotics or more of the same? *Br. J. Pharmacol.* 163 184–194. 10.1111/j.1476-5381.2011.01250.x 21323894 PMC3085877

[B21] Cook G. M. Greening C. Hards K. Berney M. (2014). Energetics of pathogenic bacteria and opportunities for drug development. *Adv. Microb. Physiol.* 65 1–62. 10.1016/bs.ampbs.2014.08.001 25476763

[B22] Cox E. Laessig K. (2014). FDA approval of bedaquiline — the benefit–risk balance for drug-resistant tuberculosis. *N. Engl. J. Med.* 371 689–691. 10.1056/nejmp1314385 25140952

[B23] Dashper S. G. Brownfield L. Slakeski N. Zilm P. S. Rogers A. H. Reynolds E. C. (2001). Sodium ion-driven serine/threonine transport in *Porphyromonas gingivalis*. *J. Bacteriol.* 183 4142–4148. 10.1128/jb.183.14.4142-4148.2001 11418553 PMC95302

[B24] Dastidar S. G. Debnath S. Mazumdar K. Ganguly K. Chakrabarty A. N. (2005). Triflupromazine: A microbicide non-antibiotic compound. *Acta Microbiol. Immunol. Hung.* 51, 75–83. 10.1556/amicr.51.2004.1-2.5 15362289

[B25] Dejon L. Speicher A. (2013). Synthesis of aurachin D and isoprenoid analogues from the myxobacterium *Stigmatella aurantiaca*. *Tetrahedron Lett.* 54 6700–6702. 10.1016/j.tetlet.2013.09.085

[B26] DePaolo-Boisvert J. A. Tuz K. Minh D. D. L. Juarez O. X. (2025). Molecular dynamics analysis of inhibitor binding interactions in the *Vibrio cholerae* respiratory complex NQR. *Proteins* doi: 10.1002/PROT.70036 [Epub ahead of print]. 10.1002/prot.70036 41017720 PMC12779168

[B27] Dibrov A. Mourin M. Dibrov P. Pierce G. N. (2022). Molecular dynamics modeling of the *Vibrio cholera* Na+-translocating NADH:quinone oxidoreductase NqrB–NqrD subunit interface. *Mol. Cell Biochem.* 477 153–165. 10.1007/s11010-021-04266-3 34626300 PMC8755685

[B28] Dibrov P. Dibrov E. Maddaford T. G. Kenneth M. Nelson J. Resch C. (2017). Development of a novel rationally designed antibiotic to inhibit a nontraditional bacterial target. *Can. J. Physiol. Pharmacol.* 95 595–603. 10.1139/cjpp-2016-0505 28425301

[B29] Dibrov P. Dibrov E. Pierce G. N. Galperin M. Y. (2004). Salt in the wound: A possible role of na^+^ gradient in chlamydial infection. *Microb. Physiol.* 8 1–6. 10.1159/000082075 15741735

[B30] Dimroth P. (1997). Primary sodium ion translocating enzymes. *Biochim. Biophys. Acta (BBA) - Bioenerget.* 1318 11–51. 10.1016/S0005-2728(96)00127-2 9030254

[B31] Elwell C. Mirrashidi K. Engel J. (2016). Chlamydia cell biology and pathogenesis. *Nat. Rev. Microbiol.* 14 385–400. 10.1038/nrmicro.2016.30 27108705 PMC4886739

[B32] Endale H. Mathewos M. Abdeta D. (2023). Potential causes of spread of antimicrobial resistance and preventive measures in one health perspective-a review. *IDR* 16 7515–7545. 10.2147/idr.s428837 38089962 PMC10715026

[B33] Engelking L. R. (2015). *Oxidative phosphorylation, in: Textbook of veterinary physiological chemistry.* Amsterdam: Elsevier, 10.1016/B978-0-12-391909-0.50036-0

[B34] Fadeeva M. S. Bertsova Y. V. Euro L. Bogachev A. V. (2011). Cys377 residue in NqrF subunit confers Ag+ sensitivity of Na+-translocating NADH:quinone oxidoreductase from *Vibrio harveyi*. *Biochem. Moscow* 76 186–195. 10.1134/S0006297911020040 21568851

[B35] Fadeeva M. S. Núñez C. Bertsova Y. V. Espín G. Bogachev A. V. (2008). Catalytic properties of Na+-translocating NADH:quinone oxidoreductases from *Vibrio harveyi*, *Klebsiella pneumoniae*, and *Azotobacter vinelandii*. *FEMS Microbiol. Lett.* 279 116–123. 10.1111/j.1574-6968.2007.01015.x 18300384

[B36] Fadeeva M. S. Yakovtseva E. A. Belevich G. A. Bertsova Y. V. Bogachev A. V. (2007). Regulation of expression of Na+ -translocating NADH:quinone oxidoreductase genes in *Vibrio harveyi* and *Klebsiella pneumoniae*. *Arch. Microbiol.* 188 341–348. 10.1007/s00203-007-0254-5 17551713

[B37] Field S. K. (2015). Bedaquiline for the treatment of multidrug-resistant tuberculosis: Great promise or disappointment? *Therapeut. Adv. Chronic Dis.* 6 170–184. 10.1177/2040622315582325 26137207 PMC4480545

[B38] Folster J. P. Katz L. McCullough A. Parsons M. B. Knipe K. Sammons S. A. (2014). Multidrug-Resistant IncA/C plasmid in *Vibrio cholerae* from Haiti. *Emerg. Infect. Dis. J.* 20 1951–1953. 10.3201/eid2011.140889 25340576 PMC4214316

[B39] Gaupp R. Schlag S. Liebeke M. Lalk M. Götz F. (2010). Advantage of upregulation of succinate dehydrogenase in *Staphylococcus aureus* biofilms. *J. Bacteriol.* 192 2385–2394. 10.1128/jb.01472-09 20207757 PMC2863491

[B40] González-Montalvo M. A. Sorescu J. M. Baltes G. Juárez O. Tuz K. (2024). The respiratory chain of *Klebsiella aerogenes* in urine-like conditions: Critical roles of NDH-2 and bd-terminal oxidases. *Front. Microbiol.* 15:1479714. 10.3389/fmicb.2024.1479714 39568993 PMC11576283

[B41] Gopal M. Padayatchi N. Metcalfe J. Z. O’Donnell M. R. (2013). Systematic review of clofazimine for the treatment of drug-resistant tuberculosis. *Int. J. Tuberc Lung. Dis.* 17 1001–1007. 10.5588/ijtld.12.0144 23541151 PMC4003893

[B42] Grauel A. Kägi J. Rasmussen T. Makarchuk I. Oppermann S. Moumbock A. F. A. (2021). Structure of *Escherichia coli* cytochrome bd-II type oxidase with bound aurachin D. *Nat. Commun.* 12:6498. 10.1038/s41467-021-26835-2 34764272 PMC8585947

[B43] Hards K. Cook G. M. (2018). Targeting bacterial energetics to produce new antimicrobials. *Drug Resistance Updates* 36 1–12. 10.1016/j.drup.2017.11.001 29499834

[B44] Häse C. C. Barquera B. (2001). Role of sodium bioenergetics in *Vibrio cholerae*. *Biochimica Biophys. Acta (BBA) - Bioenerget.* 1505 169–178. 10.1016/S0005-2728(00)00286-3 11248198

[B45] Häse C. C. Mekalanos J. J. (1998). TcpP protein is a positive regulator of virulence gene expression in *Vibrio cholerae*. *Proc. Natl. Acad. Sci. U. S. A.* 95 730–734. 10.1073/pnas.95.2.730 9435261 PMC18489

[B46] Häse C. C. Mekalanos J. J. (1999). Effects of changes in membrane sodium flux on virulence gene expression in *Vibrio cholerae*. *Proc. Natl. Acad. Sci. U. S. A.* 96 3183–3187. 10.1073/pnas.96.6.3183 10077658 PMC15916

[B47] Häse C. C. Fedorova N. D. Galperin M. Y. Dibrov P. A. (2001). Sodium ion cycle in bacterial pathogens: Evidence from cross-genome comparisons. *Microbiol. Mol. Biol. Rev.* 65 353–370. 10.1128/MMBR.65.3.353-370.2001 11528000 PMC99031

[B48] Hau J.-L. Kaltwasser S. Muras V. Casutt M. S. Vohl G. Claußen B. (2023). Conformational coupling of redox-driven Na^2^-translocation in *Vibrio cholerae* NADH:quinone oxidoreductase. *Nat. Struct. Mol. Biol.* 30 1686–1694. 10.1038/s41594-023-01099-0 37710014 PMC10643135

[B49] Hayashi M. Miyoshi T. Sato M. Unemoto T. (1992). Properties of respiratory chain-linked Na+-independent NADH-quinone reductase in a marine *Vibrio alginolyticus*. *Biochim. Biophys. Acta (BBA) - Bioenerget.* 1099 145–151. 10.1016/0005-2728(92)90211-J 1543699

[B50] Hayashi M. Shibata N. Nakayama Y. Yoshikawa K. Unemoto T. (2002). Korormicin insensitivity in *Vibrio alginolyticus* is correlated with a single point mutation of Gly-140 in the NqrB subunit of the Na+-translocating NADH-quinone reductase. *Arch. Biochem. Biophys.* 401 173–177. 10.1016/S0003-9861(02)00007-3 12054467

[B51] Heikal A. Nakatani Y. Dunn E. Weimar M. R. Day C. L. Baker E. N. (2014). Structure of the bacterial type II NADH dehydrogenase: A monotopic membrane protein with an essential role in energy generation: Structure of bacterial NDH-2. *Mol. Microbiol.* 91 950–964. 10.1111/mmi.12507 24444429

[B52] Hreha T. N. Foreman S. Duran-Pinedo A. Morris A. R. Diaz-Rodriguez P. Jones J. A. (2021). The three NADH dehydrogenases of *Pseudomonas aeruginosa*: Their roles in energy metabolism and links to virulence. *PLoS One* 16:e0244142. 10.1371/journal.pone.0244142 33534802 PMC7857637

[B53] Hu Y. Yuan M. Julian A. Tuz K. Juárez O. (2024). Identification of complex III, NQR, and SDH as primary bioenergetic enzymes during the stationary phase of *Pseudomonas aeruginosa* cultured in urine-like conditions. *Front. Microbiol.* 15:1347466. 10.3389/fmicb.2024.1347466 38468849 PMC10926992

[B54] Huai P. Li F. Chu T. Liu D. Liu J. Zhang F. (2020). Prevalence of genital *Chlamydia trachomatis* infection in the general population: A meta-analysis. *BMC Infect. Dis.* 20:589. 10.1186/s12879-020-05307-w 32770958 PMC7414538

[B55] Huda M. N. Morita Y. Kuroda T. Mizushima T. Tsuchiya T. (2001). Na+-driven multidrug efflux pump VcmA from *Vibrio cholerae* non-O1, a non-halophilic bacterium. *FEMS Microbiol. Lett.* 203 235–239. 10.1111/j.1574-6968.2001.tb10847.x 11583854

[B56] Ito T. Murai M. Ninokura S. Kitazumi Y. Mezic K. G. Cress B. F. (2017). Identification of the binding sites for ubiquinone and inhibitors in the Na+-pumping NADH-ubiquinone oxidoreductase from *Vibrio cholerae* by photoaffinity labeling. *J. Biol. Chem.* 292 7727–7742. 10.1074/jbc.M117.781393 28298441 PMC5427254

[B57] Juarez O. Barquera B. (2012). Insights into the mechanism of electron transfer and sodium translocation of the Na+-pumping NADH:quinone oxidoreductase. *Biochim. Biophys. Acta* 1817 1823–1832. 10.1016/j.bbabio.2012.03.017 22465856 PMC8172247

[B58] Juarez O. Fuller J. (2022). *Cryo EM structure of Vibrio cholerae NQR. PDB ID: 8EVU.* Piscataway, NJ: RCSB Protein Data Bank.

[B59] Juárez O. Athearn K. Gillespie P. Barquera B. (2009). Acid residues in the transmembrane helices of the Na+-pumping NADH:quinone oxidoreductase from *Vibrio cholerae* involved in sodium translocation. *Biochemistry* 48 9516–9524. 10.1021/bi900845y 19694431 PMC2758334

[B60] Juárez O. Morgan J. E. Nilges M. J. Barquera B. (2010). Energy transducing redox steps of the Na^+^ -pumping NADH:quinone oxidoreductase from *Vibrio cholerae*. *Proc. Natl. Acad. Sci. U. S. A.* 107 12505–12510. 10.1073/pnas.1002866107 20616050 PMC2906589

[B61] Juarez O. Neehaul Y. Turk E. Chahboun N. DeMicco J. M. Hellwig P. (2012). The role of glycine residues 140 and 141 of subunit B in the functional ubiquinone binding site of the Na+-pumping NADH:quinone Oxidoreductase from *Vibrio cholerae*. *J. Biol. Chem.* 287 25678–25685. 10.1074/jbc.M112.366088 22645140 PMC3408181

[B62] Juárez O. Nilges M. J. Gillespie P. Cotton J. Barquera B. (2008). Riboflavin is an active redox cofactor in the Na+-pumping NADH: Quinone oxidoreductase (Na+-NQR) from *Vibrio cholerae*. *J. Biol. Chem.* 283 33162–33167. 10.1074/jbc.M806913200 18832377 PMC2586278

[B63] Kaila V. R. I. Wikström M. (2021). Architecture of bacterial respiratory chains. *Nat Rev Microbiol* 19 319–330. 10.1038/s41579-020-00486-4 33437024

[B64] Kishikawa J. Ishikawa M. Masuya T. Murai M. Kitazumi Y. Butler N. L. (2022). Cryo-EM structures of Na^+^-pumping NADH-ubiquinone oxidoreductase from *Vibrio cholerae*. *Nat. Commun.* 13:4082. 10.1038/s41467-022-31718-1 35882843 PMC9325719

[B65] Kunze B. Höfle G. Reichenbach H. (1987). The aurachins, new quinoline antibiotics from myxobacteria: Production, physico-chemical and biological properties. *J. Antibiot.* 40 258–265. 10.7164/antibiotics.40.258 3106289

[B66] Lencina A. M. Franza T. Sullivan M. J. Ulett G. C. Ipe D. S. Gaudu P. (2018). Type 2 NADH dehydrogenase is the only point of entry for electrons into the *Streptococcus agalactiae* respiratory chain and is a potential drug target. *mBio* 9:e01034-18. 10.1128/mBio.01034-18 29970468 PMC6030563

[B67] Liang P. Fang X. Hu Y. Yuan M. Raba D. A. Ding J. (2020). The aerobic respiratory chain of *Pseudomonas aeruginosa* cultured in artificial urine media: Role of NQR and terminal oxidases. *PLoS One* 15:e0231965. 10.1371/journal.pone.0231965 32324772 PMC7179901

[B68] Liang P. Rosas-Lemus M. Patel D. Fang X. Tuz K. Juárez O. (2018). Dynamic energy dependency of *Chlamydia trachomatis* on host cell metabolism during intracellular growth: Role of sodium-based energetics in chlamydial ATP generation. *J. Biol. Chem.* 293 510–522. 10.1074/jbc.M117.797209 29123027 PMC5767857

[B69] Lin P.-C. Türk K. Häse C. C. Fritz G. Steuber J. (2007). Quinone reduction by the Na^+^ -translocating NADH dehydrogenase promotes extracellular superoxide production in *Vibrio cholerae*. *J. Bacteriol.* 189 3902–3908. 10.1128/JB.01651-06 17322313 PMC1913329

[B70] Liu Y. Tong Z. Shi J. Li R. Upton M. Wang Z. (2021). Drug repurposing for next-generation combination therapies against multidrug-resistant bacteria. *Theranostics* 11 4910–4928. 10.7150/thno.56205 33754035 PMC7978324

[B71] Manyi-Loh C. Mamphweli S. Meyer E. Okoh A. (2018). Antibiotic use in agriculture and its consequential resistance in environmental sources: Potential public health implications. *Molecules* 23:795. 10.3390/molecules23040795 29601469 PMC6017557

[B72] Marin M. A. Thompson C. C. Freitas F. S. Fonseca E. L. Aboderin A. O. Zailani S. B. (2013). Cholera outbreaks in nigeria are associated with multidrug resistant atypical El Tor and Non-O1/Non-O139 *Vibrio cholerae*. *PLoS Negl. Trop. Dis.* 7:e2049. 10.1371/journal.pntd.0002049 23459673 PMC3573102

[B73] Masuya T. Sano Y. Tanaka H. Butler N. L. Ito T. Tosaki T. (2020). Inhibitors of a Na+-pumping NADH-ubiquinone oxidoreductase play multiple roles to block enzyme function. *J. Biol. Chem.* 295 12739–12754. 10.1074/jbc.RA120.014229 32690607 PMC7476727

[B74] Mazumder R. Ganguly K. Dastidar S. G. Chakrabarty A. N. (2001). Trifluoperazine: A broad spectrum bactericide especially active on staphylococci and vibrios. *Int. J. Antimicrob. Agents* 18 403–406. 10.1016/S0924-8579(01)00324-7 11691578

[B75] Meister A. (2009). “Advances in enzymology and related areas of molecular biology,” in *Advances in enzymology*, ed. Aufl (Hoboken, NJ: Wiley).

[B76] Merrell D. S. Hava D. L. Camilli A. (2002). Identification of novel factors involved in colonization and acid tolerance of *Vibrio cholerae*. *Mol. Microbiol.* 43 1471–1491. 10.1046/j.1365-2958.2002.02857.x 11952899

[B77] Minato Y. Fassio S. R. Reddekopp R. L. Häse C. C. (2014). Inhibition of the sodium-translocating NADH-ubiquinone oxidoreductase [Na+-NQR] decreases cholera toxin production in *Vibrio cholerae* O1 at the late exponential growth phase. *Microb. Pathog* 66 36–39. 10.1016/j.micpath.2013.12.002 24361395 PMC3944667

[B78] Mo J. Si H. Liu S. Zeng Q. Cai M. Liu Z. (2023). Effect of the *Pseudomonas* metabolites HQNO on the *Toxoplasma gondii* RH strain *in vitro* and *in vivo*. *Int. J. Parasitol. Drugs Drug Resistance* 21 74–80. 10.1016/j.ijpddr.2023.02.001 36758272 PMC9929485

[B79] Mogi T. Matsushita K. Murase Y. Kawahara K. Miyoshi H. Ui H. (2009). Identification of new inhibitors for alternative NADH dehydrogenase (NDH-II). *FEMS Microbiol. Lett.* 291 157–161. 10.1111/j.1574-6968.2008.01451.x 19076229

[B80] Molnár J. Mándi Y. Király J. (1976). Antibacterial effect of some phenothiazine compounds and R-factor elimination by chlorpromazine. *Acta Microbiol. Acad. Sci. Hung* 23 45–54. 820163

[B81] Montero D. A. Vidal R. M. Velasco J. George S. Lucero Y. Gómez L. A. (2023). *Vibrio cholerae*, classification, pathogenesis, immune response, and trends in vaccine development. *Front. Med.* 10:1155751. 10.3389/fmed.2023.1155751 37215733 PMC10196187

[B82] Murray C. J. L. Ikuta K. S. Sharara F. Swetschinski L. Robles Aguilar G. Gray A. (2022). Global burden of bacterial antimicrobial resistance in 2019: A systematic analysis. *Lancet* 399 629–655. 10.1016/s0140-6736(21)02724-0 35065702 PMC8841637

[B83] Naghavi M. Vollset S. E. Ikuta K. S. Swetschinski L. R. Gray A. P. Wool E. E. (2024). Global burden of bacterial antimicrobial resistance 1990–2021: A systematic analysis with forecasts to 2050. *Lancet* 404 1199–1226. 10.1016/S0140-6736(24)01867-1 39299261 PMC11718157

[B84] Nakayama Y. Hayashi M. Yoshikawa K. Mochida K. Unemoto T. (1999). Inhibitor studies of a new antibiotic, korormicin, 2-n-Heptyl-4-hydroxyquinoline N-oxide and Ag+ toward the Na+-translocating NADH-Quinone reductase from the marine *Vibrio alginolyticus*. *Biol. Pharmaceut. Bull.* 22 1064–1067. 10.1248/bpb.22.1064 10549856

[B85] Nardulli P. Hall G. G. Quarta A. Fruscio G. Laforgia M. Garrisi V. M. (2022). Antibiotic abuse and antimicrobial resistance in hospital environment: A retrospective observational comparative study. *Medicina* 58:1257. 10.3390/medicina58091257 36143934 PMC9505554

[B86] Nossier E. S. Anwar M. M. El-Zahabi M. A. (2025). Recent advances in drug repositioning and rediscovery for different therapeutic activities utilizing updated technological approaches. *Mol. Divers.* 10.1007/s11030-025-11248-w [Epub ahead of print]. 40613877 PMC12926256

[B87] Ojeda Rodriguez J. A. Hashmi M. F. Kahwaji C. I. (2025). *Vibrio cholerae infection.* Treasure Island, FL: StatPearls. 30252355

[B88] Peyclit L. Baron S. A. Rolain J.-M. (2019). Drug repurposing to fight colistin and carbapenem-resistant bacteria. *Front. Cell. Infect. Microbiol.* 9:193. 10.3389/fcimb.2019.00193 31245302 PMC6579884

[B89] Raba D. A. Rosas-Lemus M. Menzer W. M. Li C. Fang X. Liang P. (2018). Characterization of the *Pseudomonas aeruginosa* NQR complex, a bacterial proton pump with roles in autopoisoning resistance. *J. Biol. Chem.* 293 15664–15677. 10.1074/jbc.RA118.003194 30135204 PMC6177581

[B90] Radloff M. Elamri I. Grund T. N. Witte L. F. Hohmann K. F. Nakagaki S. (2021). Short-chain aurachin D derivatives are selective inhibitors of *E. coli* cytochrome bd-I and bd-II oxidases. *Sci. Rep.* 11:23852. 10.1038/s41598-021-03288-7 34903826 PMC8668966

[B91] Reyes-Prieto A. Barquera B. Juárez O. (2014). Origin and evolution of the sodium -pumping NADH: Ubiquinone oxidoreductase. *PLoS One* 9:e96696. 10.1371/journal.pone.0096696 24809444 PMC4014512

[B92] Rijal N. Acharya J. Adhikari S. Upadhaya B. P. Shakya G. Kansakar P. (2019). Changing epidemiology and antimicrobial resistance in *Vibrio cholerae*: AMR surveillance findings (2006–2016) from Nepal. *BMC Infect. Dis.* 19:801. 10.1186/s12879-019-4432-2 31510925 PMC6739981

[B93] Salam Md. A. Al-Amin Md. Y. Salam M. T. Pawar J. S. Akhter N. Rabaan A. A. (2023). Antimicrobial resistance: A growing serious threat for global public health. *Healthcare* 11:1946. 10.3390/healthcare11131946 37444780 PMC10340576

[B94] Schurig-Briccio L. A. Parraga Solorzano P. K. Lencina A. M. Radin J. N. Chen G. Y. Sauer J. (2020). Role of respiratory NADH oxidation in the regulation of *Staphylococcus aureus* virulence. *EMBO Rep.* 21:e45832. 10.15252/embr.201845832 32202364 PMC7202225

[B95] Smith A. M. Njanpop-Lafourcade B.-M. Mengel M. A. Gessner B. D. Sauvageot D. Bidjada B. (2015). Comparative characterization of *Vibrio cholerae* O1 from five sub-saharan african countries using various phenotypic and genotypic techniques. *PLoS One* 10:e0142989. 10.1371/journal.pone.0142989 26606536 PMC4659613

[B96] Stephens R. S. Kalman S. Lammel C. Fan J. Marathe R. Aravind L. (1998). Genome sequence of an obligate intracellular pathogen of humans: *Chlamydia trachomatis*. *Science* 282 754–759. 10.1126/science.282.5389.754 9784136

[B97] Steuber J. Fritz G. (2024). The Na+-translocating NADH:quinone oxidoreductase (Na^+^-NQR): Physiological role, structure and function of a redox-driven, molecular machine. *Biochim. Biophys. Acta (BBA) - Bioenerget.* 1865:149485. 10.1016/j.bbabio.2024.149485 38955304

[B98] Steuber J. Vohl G. Casutt M. S. Vorburger T. Diederichs K. Fritz G. (2014). Structure of the *V. cholerae* Na^+^-pumping NADH:quinone oxidoreductase. *Nature* 516 62–67. 10.1038/nature14003 25471880

[B99] Steuber J. Vohl G. Muras V. Toulouse C. Claußen B. Vorburger T. (2015). The structure of Na+-translocating of NADH:ubiquinone oxidoreductase of *Vibrio cholerae*: Implications on coupling between electron transfer and Na+ transport. *Biol. Chem.* 396 1015–1030. 10.1515/hsz-2015-0128 26146127

[B100] Thanacoody H. K. R. (2007). Thioridazine: Resurrection as an antimicrobial agent? *Br. J. Clin. Pharmacol.* 64 566–574. 10.1111/j.1365-2125.2007.03021.x 17764469 PMC2203271

[B101] Thapa Shrestha U. Adhikari N. Maharjan R. Banjara M. R. Rijal K. R. Basnyat S. R. (2015). Multidrug resistant *Vibrio cholerae* O1 from clinical and environmental samples in Kathmandu city. *BMC Infect. Dis.* 15:104. 10.1186/s12879-015-0844-9 25888391 PMC4350293

[B102] Tokuda H. Unemoto T. (1984). Na+ is translocated at NADH:quinone oxidoreductase segment in the respiratory chain of *Vibrio alginolyticus*. *J. Biol. Chem.* 259 7785–7790. 10.1016/S0021-9258(17)42862-6 6736026

[B103] Torres A. Kasturiarachi N. DuPont M. Cooper V. S. Bomberger J. Zemke A. (2019). NADH dehydrogenases in *Pseudomonas aeruginosa* growth and virulence. *Front. Microbiol.* 10:75. 10.3389/fmicb.2019.00075 30804898 PMC6370648

[B104] Toulouse C. Metesch K. Pfannstiel J. Steuber J. (2018). Metabolic reprogramming of *Vibrio cholerae* impaired in respiratory NADH oxidation is accompanied by increased copper sensitivity. *J. Bacteriol.* 200:e00761-17. 10.1128/JB.00761-17 29735761 PMC6040201

[B105] Tovar-Nieto A. M. Flores-Padilla L. E. Rivas-Santiago B. Trujillo-Paez J. V. Lara-Ramirez E. E. Jacobo-Delgado Y. M. (2024). The repurposing of FDA-Approved drugs as FtsZ inhibitors against *Mycobacterium tuberculosis*: An in silico and in vitro study. *Microorganisms* 12:1505. 10.3390/microorganisms12081505 39203348 PMC11356655

[B106] Tuz K. Mezic K. G. Xu T. Barquera B. Juárez O. (2015). The kinetic reaction mechanism of the *Vibrio cholerae* sodium-dependent NADH dehydrogenase. *J. Biol. Chem.* 290 20009–20021. 10.1074/jbc.M115.658773 26004776 PMC4536408

[B107] Tuz K. Yuan M. Hu Y. Do T. T. T. Willow S. Y. DePaolo-Boisvert J. A. (2022). Identification of the riboflavin cofactor-binding site in the *Vibrio cholerae* ion-pumping NQR complex: A novel structural motif in redox enzymes. *J. Biol. Chem.* 298:102182. 10.1016/j.jbc.2022.102182 35752362 PMC9293633

[B108] U.S. Food and Drug Administration (2013). *Drug approval package information of NDA 204-384 (Sirturo^®^, bedaquiline).* Silver Spring, MD: FDA Drug Approvals and Databases.

[B109] Unemoto T. Ogura T. Hayashi M. (1993). Modifications by Na+ and K+, and the site of Ag+ inhibition in the Na+-translocating NADH-quinone reductase from a marine *Vibrio alginolyticus*. *Biochim. Biophys. Acta (BBA) - Bioenerget.* 1183 201–205. 10.1016/0005-2728(93)90019-C

[B110] Urbina F. Puhl A. C. Ekins S. (2021). Recent advances in drug repurposing using machine learning. *Curr. Opin. Chem. Biol.* 65 74–84. 10.1016/j.cbpa.2021.06.001 34274565 PMC8671152

[B111] Van Alst A. J. Demey L. M. DiRita V. J. (2022). *Vibrio cholerae* requires oxidative respiration through the bd-I and cbb3 oxidases for intestinal proliferation. *PLoS Pathog* 18:e1010102. 10.1371/journal.ppat.1010102 35500027 PMC9109917

[B112] Van Boeckel T. P. Brower C. Gilbert M. Grenfell B. T. Levin S. A. Robinson T. P. (2015). Global trends in antimicrobial use in food animals. *Proc. Natl. Acad. Sci. U. S. A.* 112 5649–5654. 10.1073/pnas.1503141112 25792457 PMC4426470

[B113] Vitt S. Prinz S. Eisinger M. Ermler U. Buckel W. (2022). Purification and structural characterization of the Na+-translocating ferredoxin: NAD+ reductase (Rnf) complex of *Clostridium tetanomorphum*. *Nat. Commun.* 13:6315. 10.1038/s41467-022-34007-z 36274063 PMC9588780

[B114] von Ballmoos C. Appoldt Y. Brunner J. Granier T. Vasella A. Dimroth P. (2002). membrane topography of the coupling ion binding site in Na+-translocating F1F0 ATP synthase. *J. Biol. Chem.* 277 3504–3510. 10.1074/jbc.M110301200 11719523

[B115] Williams-Nguyen J. Sallach J. B. Bartelt-Hunt S. Boxall A. B. Durso L. M. McLain J. E. (2016). Antibiotics and antibiotic resistance in agroecosystems: State of the science. *J. Environ. Qual.* 45 394–406. 10.2134/jeq2015.07.0336 27065386

[B116] World Health Organization (2020). *Cholera.* Geneva: World Health Organization.

[B117] World Health Organization (2024a). *2023 Antibacterial agents in clinical and preclinical development: An overview and analysis*, 1st Edn. Geneva: World Health Organization.

[B118] World Health Organization (2024b). *Bacterial priority pathogens list 2024: Bacterial pathogens of public health importance, to guide research, development, and strategies to prevent and control antimicrobial resistance*, 1st Edn. Geneva: World Health Organization.

[B119] World Health Organization (2024c). *WHO antibacterial preclinical pipeline review.* Geneva: World Health Organization.

[B120] Xi C. Zhang Y. Marrs C. F. Ye W. Simon C. Foxman B. (2009). Prevalence of antibiotic resistance in drinking water treatment and distribution systems. *Appl. Environ. Microbiol.* 75 5714–5718. 10.1128/AEM.00382-09 19581476 PMC2737933

[B121] Yoshikawa K. Nakayama Y. Hayashi M. Unemoto T. Mochida K. (1999). Korormicin, an antibiotic specific for gram-negative marine bacteria, strongly inhibits the respiratory chain-linked Na+-translocating NADH: Quinone reductase from the marine *Vibrio alginolyticus*. *J. Antibiot.* 52 182–185. 10.7164/antibiotics.52.182 10344574

[B122] Yuan M. González Montalvo M. A. Hu Y. Tuz K. Juárez O. X. (2025). Repurposing clofazimine as an antibiotic to treat cholea: Identification of cellular and structural targets. *J. Biol. Chem.* 301:110458. 10.1016/j.jbc.2025.110458 40619003 PMC12336824

[B123] Zhang Y. Marrs C. F. Simon C. Xi C. (2009). Wastewater treatment contributes to selective increase of antibiotic resistance among *Acinetobacter* spp. *Sci. Total Environ.* 407 3702–3706. 10.1016/j.scitotenv.2009.02.013 19321192

